# Genomic instability in an interspecific hybrid of the genus *Saccharomyces*: a matter of adaptability

**DOI:** 10.1099/mgen.0.000448

**Published:** 2020-10-06

**Authors:** Miguel Morard, Clara Ibáñez, Ana C. Adam, Amparo Querol, Eladio Barrio, Christina Toft

**Affiliations:** ^1^​ Departament de Genètica, Universitat de València, Burjassot, Valencia, Spain; ^2^​ Departamento de Biotecnología de los Alimentos, Instituto de Agroquímica y Tecnología de los Alimentos (IATA), CSIC, Paterna, Valencia, Spain; ^3^​ Program for Systems Biology of Molecular Interactions and Regulation, Institute for Integrative Systems Biology (I2SysBio), UV-CSIC, Valencia, Spain

**Keywords:** adaptation, genome instability, hybrids, resequencing, *Saccharomyces cerevisiae*, *Saccharomyces kudriavzevii*

## Abstract

Ancient events of polyploidy have been linked to huge evolutionary leaps in the tree of life, while increasing evidence shows that newly established polyploids have adaptive advantages in certain stress conditions compared to their relatives with a lower ploidy. The genus *Saccharomyces* is a good model for studying such events, as it contains an ancient whole-genome duplication event and many sequenced *Saccharomyces cerevisiae* are, evolutionary speaking, newly formed polyploids. Many polyploids have unstable genomes and go through large genome erosions; however, it is still unknown what mechanisms govern this reduction. Here, we sequenced and studied the natural *S. cerevisiae* × *Saccharomyces kudriavzevii* hybrid strain, VIN7, which was selected for its commercial use in the wine industry. The most singular observation is that its nuclear genome is highly unstable and drastic genomic alterations were observed in only a few generations, leading to a widening of its phenotypic landscape. To better understand what leads to the loss of certain chromosomes in the VIN7 cell population, we looked for genetic features of the genes, such as physical interactions, complex formation, epistatic interactions and stress responding genes, which could have beneficial or detrimental effects on the cell if their dosage is altered by a chromosomal copy number variation. The three chromosomes lost in our VIN7 population showed different patterns, indicating that multiple factors could explain the mechanisms behind the chromosomal loss. However, one common feature for two out of the three chromosomes is that they are among the smallest ones. We hypothesize that small chromosomes alter their copy numbers more frequently as a low number of genes is affected, meaning that it is a by-product of genome instability, which might be the chief driving force of the adaptability and genome architecture of this hybrid.

## Data Summary

1. All sequencing data generated in this study are available from the National Center for Biotechnology Information under BioProject PRJNA611499, BioSample accession number SAMN11349820, and SRA accession numbers SRR11301281 (Roche 454) and SRR9925222 (Illumina data).
2. Genetic interactions were downloaded from http://thecellmap.org/costanzo2016/.
3. Complexes were downloaded from the CYC2008 database – http://wodaklab.org/cyc2008/resources/CYC2008_complex.tab.
4. Physical protein interactions were downloaded from the *Saccharomyces* Genome Database (SGD) – https://downloads.yeastgenome.org/curation/literature/interaction_data.tab.
5. Genomes for determining chromosome copy number frequencies were downloaded from http://1002genomes.u-strasbg.fr/files/.

**Impact Statement**

Polyploidy and allopolyploidy are gaining attention as important drivers of genome evolution, more specifically, their central role in plant and microbe domestication. In yeasts, hybridization is now used in industry to improve stress resistance and different relevant characteristics of the fermentative processes. Thanks to the soar of sequencing studies, it has become evident that industrial and domesticated yeasts harbour large genomic rearrangements such as polysomies and translocations. This is also the case for allopolyploid yeast. Genomic rearrangements can be important and frequent in these strains; however, the relation between them and selective pressures are not evident. In this study, we sequenced an allotriploid strain, VIN7, and show that its genome is unstable, and this instability can widen its phenotypic landscape. Moreover, we provide evidence that the genomic changes, most frequently observed, are the mirror of a high genomic instability, which could be an advantage in industrial and changing environments, due to their more neutral effects. This study provides more evidence of the link between domestication, genomic instability and hybridization as different sides of the same process : genomic flexibility to withstand ever-changing environments.

## Introduction

Duplication of the whole genome, either by self-genome duplication or hybridization between two phylogenetically related species, also referred to as autopolyploidy and allopolyploidy, respectively, have been linked to large evolutionary leaps in all kingdoms of life, i.e. plants [1–5], vertebrates [6–8], fungi [9–11]. In the case of hybridization, this is in part due to the inheritance of traits from both parents, although seldom in an accumulative way [12–14]. The divergence between the parental species has been linked with the ploidy of newly formed hybrids, with allopolyploids tending to have more diverged parents than homoploids [15]. Another important property of increasing ploidy of the genome is redundancy, which provides genetic buffering resulting in increasing genetic robustness [16]. Niche specialization for hybrids can occur rapidly, even in a few generations [17]. Despite the evidence for the power of this evolutionary mechanism, it comes with a high risk of failure, as newly formed hybrids exhibit low fertility, small population sizes and low variability, which increases their chance of being outcompeted in stable conditions and are often considered as evolutionary dead-ends [18].

Hybridization has played an important role in the domestication of different species. Agriculturally, human pressure on crop evolution has selected, for example, fruit size, yield, taste and evenness of maturation [19, 20]. Sequencing of cultivated crops, such as wheat, strawberries, mandarins, carrots, etc., have shown that they have undergone multiple hybridizations/crossings to become the fruits we know today [21]. Likewise, in fermentative conditions, where humans use microbes/yeasts in the production of alcoholic beverages, hybrid species have been isolated [22–24]. Here, the predominant hybrids are a cross between the good fermenter *Saccharomyces cerevisiae* and one of the cold-tolerant species of the genus, such as *Saccharomyces kudriavzevii, Saccharomyces eubayanus* or *Saccharomyces uvarum* [22–24]. Inheriting properties from both parents is indicative that the newly formed hybrid is often more cold-tolerant than *S. cerevisiae* strains and more ethanol-tolerant than the other parent species, e.g. *S. kudriavzevii* [25, 26]. Therefore, hybridization is shown to be an important domestication event in the genus *Saccharomyces*.

The central role of hybridization of plants in human civilization has resulted in huge efforts in understanding the evolutionary consequences of this mechanism. It has been observed that newly formed polyploids go through large genomic changes after the initial ‘genomic shock’ of duplicating the genetic material. These changes include recombination between sub-genomes, gene/chromosomal losses and transcriptome rewiring [27–30]. The second face of hybrid evolution involves reshaping the large genomic redundancy through further gene loss or sub- and neo-functionalization of the retained genes [31, 32]. These early faces are thought to be a period of genomic instability and have been shown to vary in length, with examples spanning from hundreds to thousands of generations [33]. Long-term consequences of this can be seen in *Saccharomyces*, where approximately 30 % of the duplicated genes originated from an interspecific hybridization event, approximately 100-200 million years ago, have been retained [10, 34, 35].

The flexibility of the genomes of genus *Saccharoymces* is highlighted with the fact that they can form hybrids despite their high sequence divergence of up to 20 % [36], and very complex hybrids have been obtained, consisting of up to six parental species [37]; however, the most commonly observed number in naturally occurring hybrids is two to three parental species. As seen in plants, newly formed allopolyploid yeasts are unstable and undergo large genomic changes just after hybridization, which results in aneuploidies and chimeric chromosomes [22, 23, 38–44]. The stability of the sub-genomes is not equal, and in one study observing parental species found the least represented parent retained on average 50 % of the genome [23]; however, the range is large and the complexity of the resulting hybrids varies [22, 23, 45–49]. Furthermore, the stability of the hybrid can be influenced by environmental factors [50].

An interesting observation from looking at polyploids in *S. cerevisiae* is that they are more prone to aneuploidy than diploids or haploids [51]. In fact, in the strains sequenced to the date, aneuploidy is found in industrial strains that are often polyploid [51–53], which is also the case for allopolyploids of the genus, suggesting a link between genome stability, aneuploidy and hybridization in domestication. Furthermore, large difference of aneuploidy in a population has been coupled with phenotypic diversity, ultimately facilitating large phenotypic leaps [54, 55]. In general, it has been revealed that the genome structure is much less stable than what was classically thought, with increasing evidence that it could be an important aspect of evolution and domestication. However, there is still a lack of observations of how fast the genomic changes in unstable hybrids can occur, and if the genomic changes observed in polyploids and allopolyploids are the result of instability or are selected.

In this study, we sequenced the genome of VIN7, a natural *S. cerevisiae *× *S. kudriavzevii *allotriploid hybrid strain commercialized by Anchor Yeast as a dry yeast for winemaking. The genome of this strain was first described by comparative genomic hybridization [47] and then by sequencing in different studies [56, 57]. Intriguingly, the genome content reported was not coherent between these studies, because the origin of the strain was different: the derivative commercial dry yeast in the first study and the original strain in the other two. The differences in genome content could be the result of selective pressure or stochasticity and genome instability. We show here that the genome of VIN7 is not stable and that its instability influences the phenotype of the strain. Furthermore, we investigate different properties of the variable chromosomes that could influence the probability of a chromosome to be lost or retained and, hence, the shape of its genome architecture to illustrate the idea that the instability in itself could be the valuable trait of hybridization for industrial yeasts.

## Methods

### Yeast strains and culture media

The hybrid yeast *S. cerevisiae* × *S. kudriavzevii* VIN7 used in this study was isolated from a commercial dry yeast sample provided by Anchor Yeast. The strain was re-hydrated and grown in GPY plates (4 % glucose, 0.5 % peptone, 0.5 % yeast extract and 2 % agar) at 25 °C overnight, from which a glycerol stock was prepared and stored at −70 °C. This glycerol stock was used as starting material for culture on a GPY plate, which was grown for 48 h.

### DNA isolation

The yeast strain isolates were cultivated in GPY medium (20 g glucose l^−1^, 5 g peptone l^−1^, 5 g yeast extract l^−1^), at 25 °C for 24 h, and DNA was isolated according to standard procedures [58] where Zymolate and SDS were used for lysing the yeast cells, followed by extraction of the DNA using potassium acetate.

### Spot plate analysis for determination of strain temperature sensitivity

The spot plate technique was used to examine the effect of temperature on the growth of the three sup-populations of VIN7 (dry yeast, glycerol stock and plate) on GPY agar. Starter yeast cultures were obtained by inoculating the yeast strains into 10 ml GPY medium and incubating overnight at 25 °C. Tubes were centrifuged at 4000 r.p.m. for 5 min. The supernatant was discarded and pellets were re-suspended in sterilized water. Cell suspensions were then prepared based on optical density determined using a spectrophotometer. Samples were diluted until an optical density of 1 was obtained at a wavelength of 600 nm. Subsequent 1 : 10 dilutions were carried out to prepare serially diluted samples. A 10 µl volume of each dilution was spotted onto GPY agar plates in triplicate. The plates were then incubated in a static incubator at 12 and 28 °C for 7 days. Data was recorded by photographing the spot plates.

### Ethanol tolerance growth analysis

Ethanol tolerance of the three sub-populations of VIN7 (dry yeast, glycerol stock and plate) was evaluated by performing growth essays in GPY medium with 10 % (v/v) ethanol. Growth was monitored by measuring the optical density at 600 nm in a SPECTROstar Omega instrument (BMG Labtech). Measurements were taken every 30 min for 43 h after a 20 s pre-shaking for all the experiments. All the experiments were carried out in sextuplicate. Growth parameters like starting optical density, maximum optical density, growth rate and area under the curve were calculated using the R package's Growthcurver [59].

### Genome sequencing, assembly and annotation

The *S. cerevisiae* × *S. kudriavzevii* VIN7 genome was sequenced using 454 technology (shotgun with 550 bp read length and paired-end reads with 8 kb insert size) in combination with paired-end Illumina technology (600 bp insert size) on a HiSeq 2000 instrument. The Illumina reads were filtered using Sickle [60] with a minimum read length of 80 bp and minimum quality score of 30. A subset of 5000000 paired-end reads was used for the *de novo* assembly, giving a coverage of 22× on the haploid genome. The program sff_extract was used for extracting the 454 reads and to clip ends with low quality and/or adaptor sequence. The coverage of the 454 reads was 1.7× for the 157000 paired-ends and 7× for the 157000shotgun reads for the haploid genome. A *de novo* assembly was carried out using mira v 3.4.1.1 (https://sourceforge.net/projects/mira-assembler/) and GS *De Novo* Assembler (Roche/454 Life Sciences). The resulting assemblies were corrected and manually edited using Consed [61], contigs were concatenated or broken based on paired-end information. More specifically, fake reads generated from the corrected GS *De Novo* Assembler assembly (run with default parameters for heterozygotic mode and minimum read length of 25) were added to the main mira assembly (run without separating out long repeats and uniform read distribution). The determination of the recombination points was done manually though Consed. The scaffolds were aligned to *S. cerevisiae* reference strain S288C and *S. kudriavzevii* IFO 1802 with the all genome aligner MUMmer [62], and with this information the scaffolds were ordered into chromosome structure using an in-house script (https://github.com/evosysmicro/LabThings/blob/master/scripts/ultraScaf.pl).

Illumina reads mappings were done on the reference genomes of the *S. cerevisiae* S288C and *S. kudriavzevii* IFO 1802 strains by using bowtie2 [63] with default settings. Any potential secondary mappings were filtered away with Samtools [64], such that only uniquely mapped reads were kept for further analysis.

The annotation was carried out in three steps. (i) Annotation from the previously published VIN7 genome (pubVIN7) [56] was transferred to the new assembly using ratt [65]. (ii) *Ab initio* gene prediction was performed with Augustus [66] to detect possible unannotated genes in the pubVIN7. (iii) The result from the two first steps was carefully and manually checked using Artemis [67] and the pubVIN7 re-annotated as we detected two principal problems in the previous annotation: (a) the presence of indels (a usual problem with 454 sequencing technology) resulted in many coding sequences (CDS) being removed; (b) genes containing introns were not annotated previously or only one of the exons was present.

### Quantitative real-time PCR detection

DNA from all yeast strains, hybrid VIN7, *S. cerevisiae* S288C and *S. kudriavzevii* CR85, was extracted following a previously described procedure [58]. DNA concentration and purity were determined with a NanoDrop ND1000 spectrophotometer (Thermo Fisher Scientific) and genomic DNA integrity was checked by electrophoresis in 0.8 % agarose gel.

Oligonucleotide primers for quantitative real-time PCR (Isogen Life Science) were species-specific and located in different chromosomes (III, VI, XI). We checked each chromosome with two pairs of primers (see Table 1) designed using the comparison between sequences from S288C and CR85 and the pubVIN7 [56].

**Table 1.** Primers used for quantitative real-time PCR

Species	Chromosome	Sequence	Forward primer (5′→3′)	Length (bp)	Reverse primer (5′→3′)	Length (bp)	Size (bp)
*S. kudriavzevii*	III	VIN7_5911	ACCTCTTCTCAAGGCTTGGC	20	AGGGGTGCATTTAGAATGGGA	21	119
		VIN7_5888	TGCGTCTTGTGCCAGTTGTA	20	GGAGAGCAGGTCAGGGTAGA	20	151
	VI	VIN7_6908	AAACGACGTATGCCGCAATG	20	CGCCTGATGAGAACCCTGTT	20	101
		VIN7_6891	ACGAAGAGAGAAAGCGTCAGG	21	AGCTGGCTCGACTTCTTCAC	20	102
	XI	VIN7_8307	ATTGGATATCCCTCCGGCAC	20	CTGCAACCATCACAATGCCC	20	101
		VIN7_8382	GATCCTATCACTGGCGCGAT	20	TCTGGCAAGTCCTTGGTGTG	20	150
*S. cerevisiae*	III	VIN7_0475	TGCTACGGTGGTTCTGCAAG	20	ACCACTGTGTCATCCGTTCT	20	152
		VIN7_0509	TAATGGAGAGCTTCATGTCGGG	22	CCCTCAAGGATGTCACTAGCA	21	106
	VI	VIN7_1475	AGCATCCAGGGATTCTCACG	20	TCCAGTATCTTGGCCGATGTG	21	199
		VIN7_1573	ACACCGCCAAGCTTCCAATA	20	TTGCCACGCAAAGAAAGGAC	20	143
	XI	VIN7_2927	CTCTAGAACAGGCTGAGGCG	20	GGTCGTATGCCTGTAGACGG	20	129
		VIN7_2979	GCAACGGGCAAAAGCAAGAT	20	ACCACCTTCCCATTTCGGTC	20	124

Real-time PCR reactions were performed in triplicate for each sample, using a LightCycler 480 SYBR Green I master kit (Roche Applied Science) and a LightCycler 480 system, following the manufacturer's instructions. The final primer concentration was 300 nM in a 10 µl reaction mixture. The amount of DNA used as a template was 20 ng, and the amplification program had 40 cycles. All the amplicons had a size between 100 and 200 bp to ensure maximal PCR efficiency. We included positive and negative controls of amplifications in all plates and previously tested all primers to discard cross-specificity. Data were analysed using the AbsQuant/2ndDerivative-Max function of the LightCycler 480 software. *C*_p_ values found for the sequences from the two strains used as positive controls were between 18 and 20. *C*_p_ values higher than 35 were considered as no amplification in our 40 cycle program.

### Interaction and expression data analyses

Data from physical interactions were extracted from the *Saccharomyces* Genome Database (SGD) interaction dataset (https://downloads.yeastgenome.org/curation/literature/interaction_data.tab). All rows labelled as physical interaction were used to perform the analysis. Genetic interactions were downloaded from http://thecellmap.org/costanzo2016/ [68] and information on protein complexes were taken from [69]. Expression data describing the response of changing the carbon source to ethanol, lactate or glycerol or oxidative stress + dextrose, compared to glucose, were extracted from the supplemental material of a published paper [70] (National Center for Biotechnology Information SRA accession no. SRP074821). Each gene was given the value 1 if it was up-regulated in the stress condition compared to the control condition, and 0 if it was either not deferentially expressed or down-regulated. To calculate the number of up-regulated genes per chromosome, gene values for each chromosome were summed.

Statistical analyses of interactions and expression per chromosome were performed using a bootstrap analysis strategy. First, the number of genes per chromosome was calculated from the *S. cerevisiae* S288C genome. To create a normal distribution, random genes from the genome were picked without replacement to create a set the same size as the chromosome. The mean number of interactions or up-regulated genes was calculated for this random chromosome. This operation was repeated 10 000 times. Then, the actual mean interactions or up-regulated genes were calculated and the *pnorm* function in R was used to calculate the cumulative distribution function (CDF) at the actual mean considering the null distribution created.

A genetic interaction network between chromosomes was created with the Costanzo *et al*. (2016) [68] data. The interactions between genes of different chromosomes were summed. We considered that positive interactions would compensate negative ones. In this way, we get a value of the overall epistatic interaction between each chromosome pair. A network with the chromosomes as nodes was then drawn. An arrow would go from one chromosome to the other if the sum of interactions between them was the nearest to 0, in other words, pointing to the chromosome with the lowest epistatic effect if the copy number of the chromosome changes.

## Results

### Genome of VIN7

**Fig. 1.**
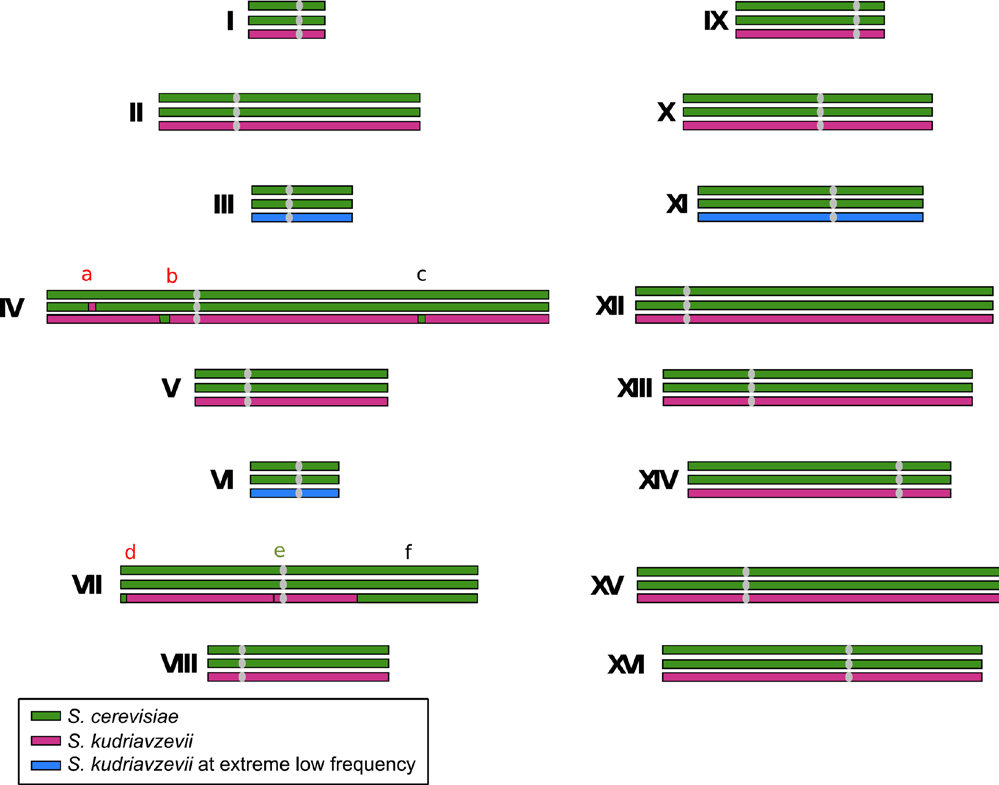
The genome structure of VIN7. The *S. cerevisiae* sub-genome is represented in green and *S. kudriavzevii* in pink; the chromosomes that are lost or are present in low frequency are shown in blue (see Fig. S2). Homeologous recombination sites are marked with letters and colour coded according to what study they were observed in: red, observed in our study and Borneman *et al*. [56, 57]; green, observed in our study but could not be confirmed in Borneman *et al*. [56, 57] as it was between contigs; black, only observed in our study. Due to the technique used by Peris *et al*. [47], they were only able to observe event f.

The wine yeast VIN7, obtained from a commercial dry yeast sample provided by Anchor Yeast, was sequenced by a combination of paired-end and shotgun sequencing with 454 and Illumina equipment. The read assembly yielded 206 contigs with an N50 of 295 173 bp and an N90 of 82 169 bp, with a total size of 21.5 Mbp, which were distributed in 106 scaffolds. According to the genome sequencing, VIN7 is an allotriploid hybrid, diploid for the *S. cerevisiae* subgenome and haploid for the *S. kudriavzevii* one, but with certain genome rearrangements and chromosome losses (Figs 1, S1 and S2, available with the online version of this article).

We detected six recombination events between two *S. kudriavzevii* and *S. cerevisiae* homeologous chromosomes, three of them corresponding to chromosome IV and the other three located in chromosome VII. One of these events involves the substitution of *S. cerevisiae* genes by their *S. kudriavzevii* orthologues, it corresponds to a 13 kb region located on chromosome IV, between the genes YDL186W and DLD2/YDL178W (event a in Fig. 1). The rest of the events are substitutions of the *S. kudriavzevii* part by the *S. cerevisiae* sequence. Two events involved chromosome IV: a 15 kb region located between genes NAT1/YDL040C and DBP10/YDL031W (event b in Fig. 1), and a 12 kb fragment located on the other arm of the chromosome, between genes FCF1/YDR339C and HXT3/YDR345C (event c in Fig. 1). The first recombination on chromosome VII involves 26 kb from the beginning of the left arm to gene HXK2/YGL253W (event d in Fig. 1). In the middle part of the gene PMA1/YGL008C, a tiny replacement of 1788 bp is observed (event e in Fig. 1). Finally, the recombination involving the largest region (~350 kb) of *S. kudriavzevii* genes replaced by their *S. cerevisiae* homologues is located on the right arm of chromosome VII, from gene SPT6/YGR116W to the end of the chromosome (event f in Fig. 1).

In most of the genome, we found contigs from both *S. cerevisiae* and *S. kudriavzevii* sub-genomes, which is consistent with an allotriploid hybrid (Fig. S1). Nevertheless, in the assembly, no contig from chromosomes III, VI and XI of the *S. kudriavzevii* sub-genome were obtained. Mapping the Illumina reads on these three chromosomes from the reference genome of *S. kudriavzevii* IFO1802 gave mean coverages of 5.6×, 10.4× and 2.5× for chromosomes III, VI and XI, respectively (Fig. S2). The Illumina library contains 116 062 916 pairs of 100 bp reads. Considering a complete triploid genome containing the genomes of both parental species, the expected mean coverage would be of 645× per haploid sub-genome. The coverage obtained for these chromosomes is clearly lower than expected, but a deeper sequencing of the VIN7 genome allowed us to find sequences from them, indicating that cells containing these chromosomes are present at a low frequency in the population.

### Our VIN7 genome differs from those previously described

**Fig. 2.**
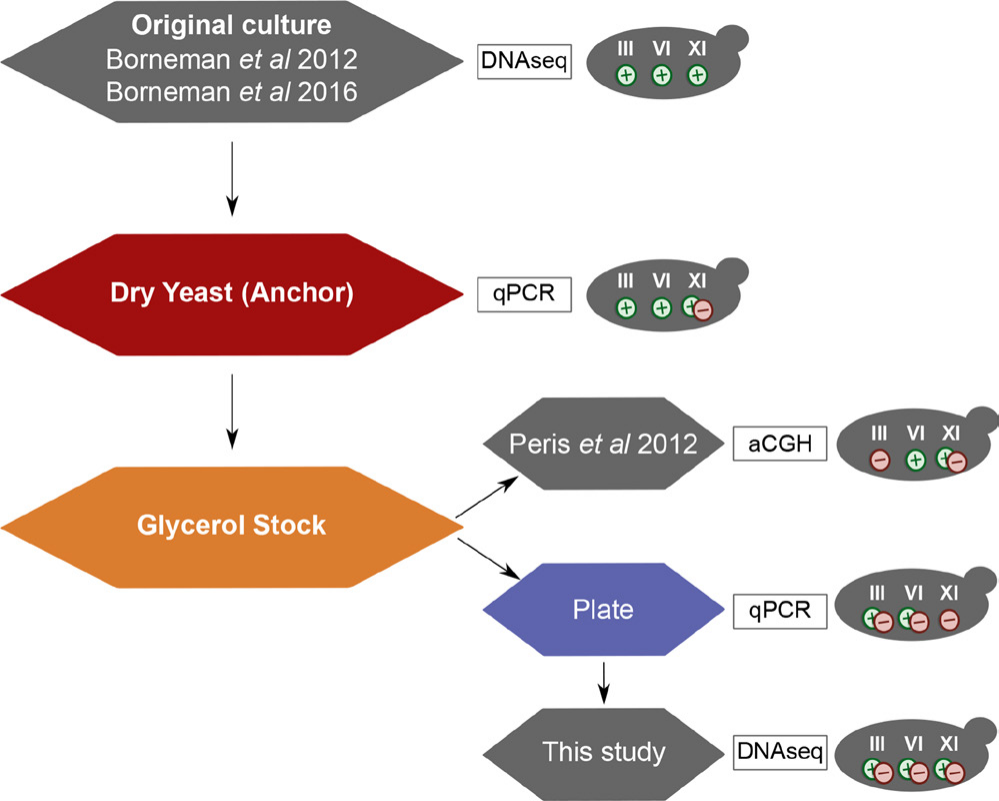
Different sub-populations of VIN7 have different chromosomal losses. Representation of the observed differences in the presence/absence of chromosomes III, VI and XI in the different publications of VIN7. +, Present; −, absent; +/–, low frequency or dubious. qPCR, Quantitative real-time PCR.

As summarized in Fig. 2, different studies described different genome structures. Borneman *et al*. [56] published the sequence of the VIN7 genome from the original culture, which was determined using 454 technology. In this assembly, the homeologous recombination in the right arm of chromosome VII is not detected. Interestingly, the rest of the recombination events are present. Moreover, the chromosomal losses described above are not observed in this assembly. In another work by the same group [57], the VIN7 genome was sequenced with Illumina technology. In this study, the authors confirmed the genome structure described in their first article [56], VIN7 being a perfect allotriploid without the chromosome VII recombination.

An array comparative genomic hybridization (aCGH) experiment [47] also reported the genome structure of VIN7. In this study, the hybrid VIN7 was obtained, as in the present study, from a sample of the commercial dry yeast provided by Anchor Yeast. In agreement with our results, the right arm of chromosome VII was present in three *S. cerevisiae* copies. The loss of the *S. kudriavzevii* chromosome III was also reported; however, *S. kudriavzevii* chromosomes VI and XI were detected in the aCGH experiment. This is despite both our sequencing and the aCGH experiment being based on the same glycerol stock. The different genome structures in the different samples indicates that the genome of VIN7 is particularly dynamic.

### VIN7 genome content is not stable and affects its phenotype

As different genome content was observed in the VIN7 strain depending on the source used to sequence it, the next question to answer was whether we could observe changes in chromosome loss in growing colonies. We performed quantitative real-time PCR analysis for chromosomes III, VI and XI of *S. cerevisiae* and *S. kudriavzevii*. Two genes from each chromosome, one from each arm, were chosen to detect its presence. Two different sub-populations were tested. The re-hydrated commercial dry yeast grown on a GPY plate at 25 °C, and a glycerol stock of this stored at −70 °C and plated at 25 °C for 48 h.

We used species-specific primers to detect by PCR the presence of chromosomes III, VI and XI. All of them were amplified except for one of the *S. cerevisiae* sequences of chromosome XI that did not amplify in any sample (Tables 2 and S1). The dry yeast showed amplification of all three chromosomes, except for *S. kudriavzevii* chromosome XI. This one seemed harder to detect and was probably lost in most of the cells. For the sub-population from the glycerol stock, grown for a couple of generations, all three *S. kudriavzevii* chromosomes were difficult to detect. *S. kudriavzevii* chromosome XI was not detected at all, indicating that it was definitely lost in the overall population. The other two *S. kudriavzevii* chromosomes were not completely lost but undetectable, as in the dry yeast sample. Altogether, these results indicate that the genome content of VIN7 is dynamic and that chromosome loss occurred in a few generations.

**Table 2.** Quantitative real-time PCR amplification results, including for the GPY plate with negative controls See Table S1 for *C*_t_ values. +, Positive amplification with 20 ng DNA; −, no amplification; +/−, amplification occurred with more than 20 ng DNA.

Chromosome	Species	Seqence	S288c	CR 85	VIN7 dry yeast	VIN7 plate
III	*S. cerevisiae*	0475	+	−	+	+
		0509	−	−	+	+
	*S. kudriavzevii*	5888	−	+	+	+/−
		5911	−	+	+	+/−
VI	*S. cerevisiae*	1475	−	−	+	+
		1573	+	−	+	+
	*S. kudriavzevii*	6891	−	+	+	+/−
		6908	−	+	+	+/−
XI	*S. cerevisiae*	2927	−	−	−	−
		2979	+	−	+	+
	*S. kudriavzevii*	8307	−	+	−	−
		8382	−	+	+/−	−

**Fig. 3.**
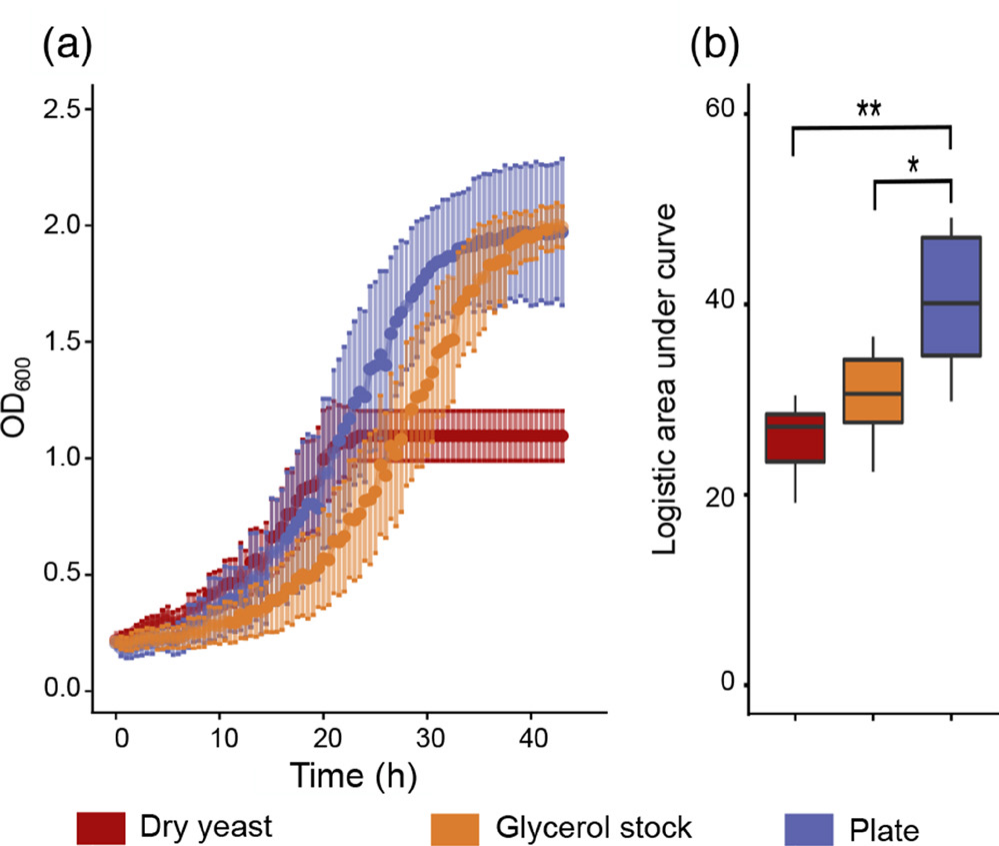
Phenotypic characterization of the different sub-populations of VIN7 on ethanol. (a) Growth cures for the dry yeast, the glycerol stock and the plate sub-population plotted as the mean OD_600_ value for each of the time points, including the standard deviation. (b) Area under the curve of the three sub-populations. Significant differences of each growth parameter are indicated as * and **, when the probabilities are *P* <0.05 and *P* <0.005, respectively, using a Wilcoxon rank test.

Genomic instability could have a potential impact on the phenotype of the strain. Here, we tested two relevant growth conditions for *Saccharomyces* hybrids to look for the potential adaptive value of the chromosomal loss. Firstly, growth at low temperature is thought to be one of the advantages that the *S. kudriavzevii* sub-genome confers to the hybrids. Using a spot assay, we did not see any differences in the growth of the VIN7 sub-populations (Fig. S3). The three different populations showed similar growth at 28 °C and also similar phenotypes at 12 °C. Secondly, growth under stress with 10 % ethanol was tested. Looking at an overall comparison between the three VIN7 populations, we observed that the plate sub-population grew significantly better than the other two when using the area under the curve as a measure of growth (Fig. 3). This indicates that the population with the lowest polymorphism of the three chromosomes is more tolerant to ethanol than the other two.

The main difference between the plate sub-population and the other two is the almost complete loss of chromosome XI in the plate sub-population. To determine whether this chromosome harbours any genes that could explain the difference in phenotype, a Gene Ontology enrichment analysis was performed on chromosome XI; however, no enrichments were observed. Two central pathways involved in the production and uptake of ethanol are the glycolysis pathway and tricarboxylic acid (TCA) cycle. Chromosome XI contains four genes in each – *GPM1*, *FBA1*, *PCK1* and *PGM1* in the glycolysis pathway, and *MDH*, *SDH1*, *SDH3* and *PCK1* in the TCA cycle – neither of these seven genes are located in a central role of the two pathways, so cannot by their own explain the advantage the plate yeast has over the other two sub-populations when grown in ethanol.

### Aneuploidy pattern analysis

**Fig. 4.**
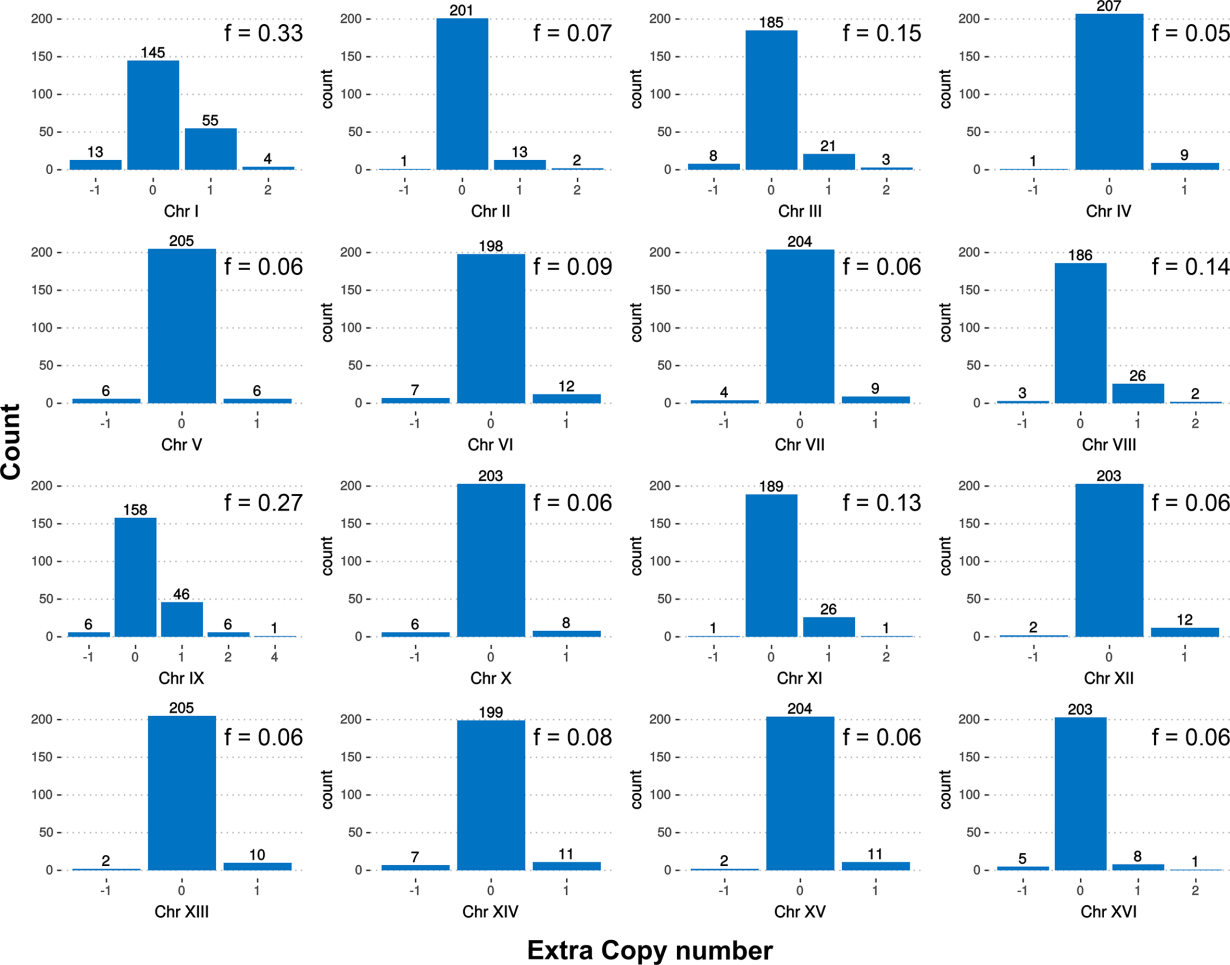
Aneuploidy frequency of the chromosomes in *S. cerevisiae*. For each chromosome, we counted the number of strains in the work by Peter *et al*. [51] that had euploid (0) or different copy numbers. The frequency was calculated as the number of strains harbouring an abnormal copy number (independently of the gain or loss) divided by the number of strains harbouring a normal copy number of each chromosome.

Many industrial *S. cerevisiae* strains show aneuploidies with adaptive potential [71]. Here, we observed a more complex genotype. We asked whether the chromosomes themselves could harbour certain traits that could explain why these were lacking in VIN7. The first question is whether certain chromosomes are more frequently aneuploids than others. As not a sufficient number of *S. cerevisiae* × *S. kudriavzevii* hybrid genomes were available, we used *S. cerevisiae* as a model. One thousand and eleven genomes of *S. cerevisiae* have been sequenced recently [51] and in this dataset 217 *S. cerevisiae* strains were aneuploids for at least one chromosome. The aneuploid frequency of each chromosome was calculated as the frequency of the appearance of abnormal chromosome copies in the aneuploid strains (Fig. 4). As previously described, having an extra copy of a chromosome compared to the overall ploidy of the genome is more frequent than losing a copy of it [51]. Interestingly, all chromosomes showed that they harbour the ability to be aneuploid, but the frequency of aneuploidy varied with a magnitude of almost seven between the chromosome with the highest aneuploidy compared the one with the lowest. The more frequently aneuploid chromosomes from our analysis were I (*f*=0.33), IX (*f*=0.27), III (*f*=0.15), VIII (*f*=0.14) and XI (*f*=0.13), with IV (*f*=0.05) being the chromosome with the lowest frequency. In line with this, the chromosomes lost in VIN7 are among the frequently aneuploid ones, except chromosome VI, which has a frequency of 0.06.

Chromosomes have different chances of being present in an abnormal copy number. This could be due to different characteristics of the genes present within those chromosomes, which could influence the possible detrimental or beneficial effect of aneuploidy. Chromosome copy number changes unbalance the content of the proteins encoded by the genes located in these chromosomes, i.e. the higher the copy number, the higher the expression. Although it has been shown that a small proportion of yeast genes show dosage compensation, they show a lower expression than expected according to their copy numbers [72]. Several characteristics could be important to select or relax selection against aneuploidy. The genomes of *S. cerevisiae* and *S. kudriavzevii* are in general collinear and gene content is largely overlapping. Furthermore, the first indication of genetic compatibility has shown that macromolecular complexes between proteins from the different sub-genomes can be formed but at a very low frequency [73, 74], meaning that the two sub-genomes act largely as two separate entities. With these properties and with the fact that very little data is available for hybrids, *S. cerevisiae* makes a good proxy for the hybrids. Here, we looked at several characteristics of the genes that could influence the chromosomal dynamics. First, physical interactions between proteins are essential for the cell: changing the protein content of interacting partners can be detrimental and, therefore, needs to be regulated, and could be an important negative selection factor against aneuploidy. Second, proteins involved in protein complexes are often dosage-sensitive: changing their copy numbers could be detrimental. Third, epistasis is another factor to be considered. If a chromosome contains highly connected genes in the interaction network, the effect of aneuploidy could be higher. However, if the genes in the chromosome are less connected, the rewiring of the interaction network would be more tolerated. Fourth, a possible positive effect of changing the chromosome copy number could be due to an increase in the dose of genes involved in stress response. We looked at different RNA-seq experiments to search for the up-regulated genes under four stresses: lactic acid, ethanol, glycerol and oxaloacetate.

**Fig. 5.**
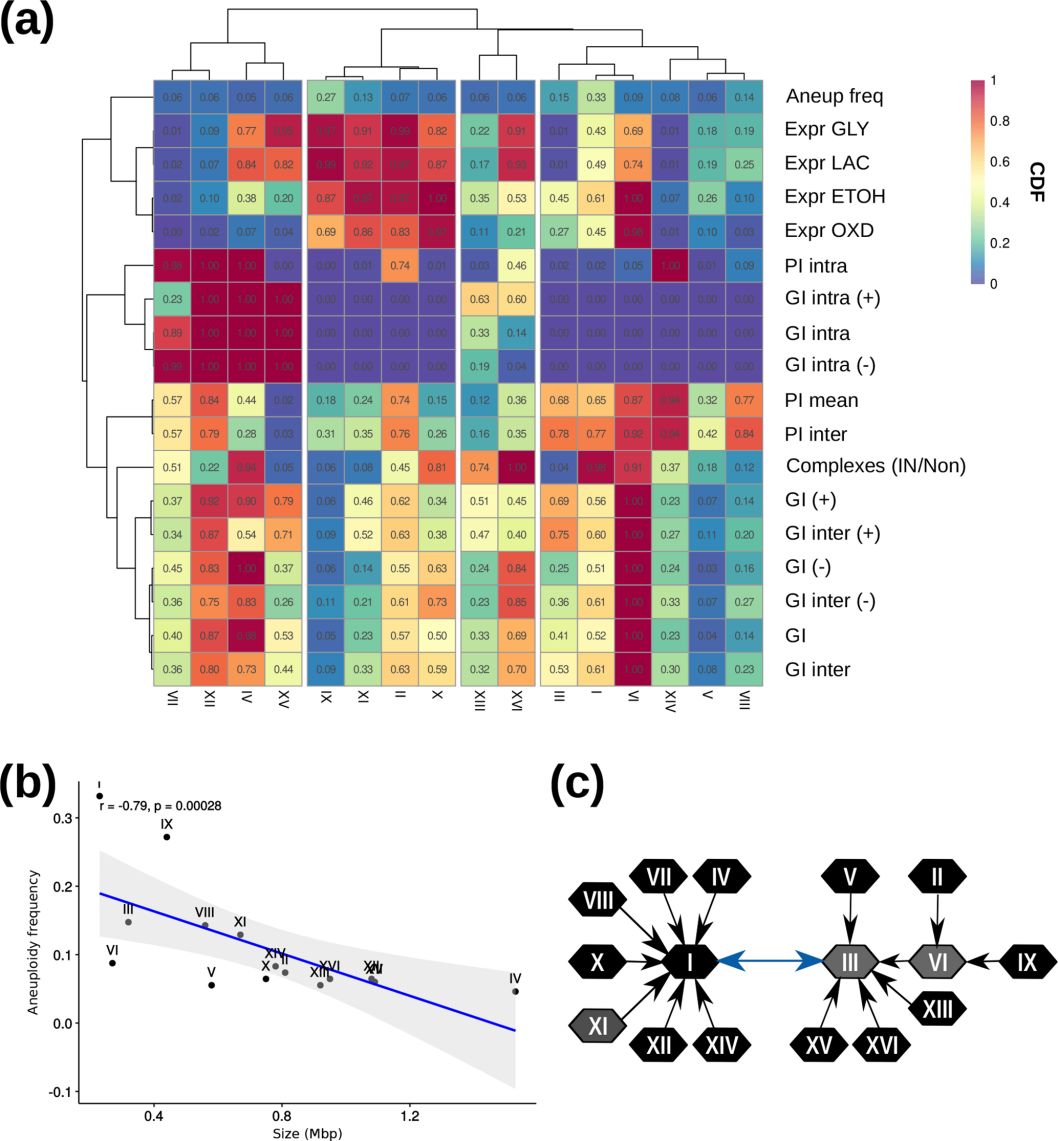
Factors affecting chromosome aneuploidy. (a) CDF value heatmap of the characteristics analysed for each chromosome and its aneuploidy frequency. Values near to one indicate a higher than expected mean and near to zero a lower than expected mean. Aneup freq, aneuploidy frequency; PI intra, intra-chromosomal physical interactions; GI intra (*+*), positive intra-chromosomal genetic interactions; GI intra, intra-chromosomal genetic interactions; GI intra (−), negative intra-chromosomal genetic interactions; Expr GLY, expression on glycerol; Expr LAC, expression on lactate; Expr ETOH, expression on ethanol; Expr OXD, expression on oxaloacetate; PI mean, mean physical interactions; PI inter, inter-chromosomal physical interactions; GI (*+*), positive genetic interactions; GI inter (*+*), positive inter-chromosomal genetic interactions; GI, genetic interactions; GI (−), negative genetic interactions; GI inter, inter-chromosomal genetic interactions; GI inter (−), negative inter-chromosomal genetic interactions; Complexes (IN/Non), proteins being in a complex or not. (b) Aneuploidy frequency of the chromosomes compared to the chromosome size. (c) Less interacting chromosomes network. The arrow between chromosomes represents the chromosome with which the interaction tends to be neutral.

To decipher whether a pattern was recurrent in the chromosomes, we performed a bootstrap analysis by generating a null distribution for each of the chromosomes. We calculated the actual mean number of interactions or up-regulated genes for each stress condition and then calculated the integral of the distribution at the left of the actual value. This value is low if the actual mean is lower than that expected by chance, and tends towards one if it is higher than expected. The heatmap in Fig. 5a depicts all the features analysed for each chromosome, mapping out the value of the CDF and using a correlation clustering to normalize the variables across chromosomes. It is worth noting that all the features analysed have a mean that is significantly lower or higher than expected in at least one chromosome and, likewise, each chromosome has at least one feature which is significantly lower or higher than expected. Interestingly, when looking at the features per chromosome, none of the interactions or stress responses seem to be clearly associated with the aneuploidy frequency. Furthermore, chromosomes III, VI and XI have different contents that could be of relevance for their losses in VIN7, but are not shared among them.

Chromosome size is another possible factor influencing the tolerance of aneuploidy. If copy number changes affect smaller chromosomes, the effects on the number of proteins, the DNA content or the gene interactions are smaller. We found a negative correlation between chromosome size and aneuploidy frequency (*r*=−0.79, *P*=0.00028; Fig. 5b). In VIN7, chromosomes III and VI are lost, and also are among the smallest chromosomes. Interestingly, chromosome VI is not in the most frequently aneuploid even if it is one of the smallest, pointing out that different characteristics, or a combination of them, can be important.

To have different aneuploidies at the same time is a complex genotype. We asked whether interactions between chromosomes could explain whether certain chromosomes could appear in abnormal numbers together, like those seen in VIN7. To do so, we calculated the number of interactions between genes of each chromosome. For each chromosome, we identified which other chromosomes it had least epistatic relationships with, with positive epistasis counterbalancing negative epistasis and vice versa. With this information, we drew a network in which each node is a chromosome and a directed arrow pointed to its least interacting partner (Fig. 5c). An interesting pattern emerged, with the network showing two principal hubs, the first one centred on chromosome I and the second one on chromosome III. Moreover, chromosome I and III are connected in both directions as one is the least interacting with the other and vice versa. Chromosome VI is connected to chromosome III, but it is also a hub of the network. The chromosomes at the centre are the less connected in the epistatic network, which would mean that the epistatic effects of removing these chromosomes would have the least detrimental effect on the cell. It is noteworthy that two of the three aneuploid chromosomes in VIN7 are central hubs of the network. Furthermore, both chromosomes I and III are frequently aneuploids in *S. cerevisiae* strains.

## Discussion

Hybridization in the genus *Saccharomyces* is recurrent in industrial environments [22, 23, 75]. As hybrids inherit properties from both parental species, this means that this phenomenon is increasingly used by companies to improve the characteristics of wines and beers. However, the genome evolution of hybrids is still an open question. Here, we sequenced the genome of the commercial *S. cerevisiae* × *S. kudriavzevii* hybrid VIN7 to decipher its genome structure and dynamics.

The VIN7 genome has been reported several times in different publications, first using comparative genomic hybridization [47] and then with two different sequencing technologies [56, 57]. All agreed that VIN7 is allotriploid with two copies of *S. cerevisiae* and one of the *S. kudriavzevii*, which cohered with what we observe here. However, two differences were detected between the sequencing experiments and the comparative genomic hybridization analysis. In the work by Peris *et al*. [47], part of the *S. kudriavzevii* chromosome VII was replaced by *S. cerevisiae*, and *S. kudriavzevii* chromosome III was lost compared to the results of Borneman *et al* [56, 57]. We observed more differences in our genome compared to what was reported in the other experiments. In addition to what was found in the work of Peris *et al*. [47], and using a derivative population from the same glycerol stock, *S. kudriavzevii* chromosomes VI and XI were apparently lost in the majority of our sequenced sample populations. Moreover, we could determine that the changes in the karyotype of the VIN7 occurred in a short number of generations. This indicates that VIN7 has a highly dynamic genome.

Here, we looked at whether the different aneuploid variants of VIN7 presented differences in phenotypic traits. Despite only two populations being tested, which were only a few generations apart from each other, we observed that ethanol tolerance was different between them, confirming that phenotype could change with the genomic content and in very few generations. In agreement with this, it has been shown in *S. cerevisiae* that different aneuploidies could change the phenotype of strains and be of relevance for industry [71]. In hybrids, a study that analysed karyotypic variants of a *Saccharomyces pastorianus* strain, a hybrid between *S. cerevisiae* and *S. eubayanus*, showed that these brewing hybrids also have a dynamic genome [39]. Interestingly, they found that these variants also showed different phenotypic characteristics. Similar results have been observed in a study with artificial *S. cerevisiae* × *S. eubayanus* hybrids, in which the authors suggested that the general ploidy of hybrids could affect their brewing fermentation characteristics as well as the production of flavour compounds [76]. These shreds of evidence point to two important features. On one hand, they emphasize the importance of the genome stability of the strains in the possible outcome of the industrial fermentation in which they are involved. If changes can occur in so few generations, it is of relevance to further study what the trigger factors could be and the connection to the stability in the final product characteristics. On the other hand, they suggest that it is of interest to determine the role of the presence of a dynamic genome in the adaptation of yeasts to industrial environments.

Aneuploidy in yeast has different effects on the fitness of the population. Aneuploidy can dramatically slow down growth; hence, decreasing cell fitness in certain conditions [77]. However, there is also evidence indicating that aneuploidy could be advantageous under stressful conditions. Chromosome polysomy increases ethanol tolerance [78], and resistance to heat stress [79] or other stresses present in industrial processes [71]. We investigated the frequency of aneuploidy for each chromosome in a large dataset of *S. cerevisiae* strains [51] (http://1002genomes.u-strasbg.fr/files/). Furthermore, we looked at different features of the gene content of the chromosomes and could observe, as other authors did [51, 53], that chromosome size is the most important factor to explain aneuploidy frequencies. Other characteristics, such as physical interactions, epistasis or stress response, are not relevant to explain the aneuploidy frequency. Therefore, we hypothesize that the higher frequency of the smallest chromosomes is due to its lower effect on DNA replication stress and on the number of genes affected. If this is so, then the fact that these are observed more frequently is a by-product of what is important in these strains: genome instability.

With genome instability, complex patterns of aneuploidy can arise. It has been shown in cells with induced chromosomal instability that the emerging pattern of chromosomal aneuploidy is not random, but governed by interactions between chromosomes [80]. This is supported by other studies [77], in which chromosome VI aneuploidy was more stable if it appeared with other aneuploidies. Zhu *et al*. [81] also showed that a specific karyotype can lead to highly unstable genomes, while others produce relatively stable ones. We found that chromosomes I and III have the least epistatic impact on the rest of chromosomes, which could be an explanation for their higher aneuploidy frequency. These would be less detrimental and, hence, more frequent in aneuploid populations, but not necessarily a signal of adaptive selection. However, although the presence of aneuploidies would be stochastic due to its neutral or slightly deleterious effect, under changing environmental conditions, such as those occurring in industrial processes, the genetic variability due to a higher genome instability could become advantageous and more frequent, because changes in gene dose would allow a faster adaptation.

Both hybrid and aneuploid strains appear more frequently in industrial environments than in nature [53]. Moreover, higher ploidies are more frequent in these environments than in ‘natural’ ones [51] and aneuploidies are more tolerated in polyploids than in lower ploidies. A recent study claims that both hybridization and aneuploidy are adaptation mechanisms to perturbed environments [53]. Here, we have presented a case study of an allopolyploid strain with an unstable genome that broadens its phenotypic space within a very short evolutionary time. In conclusion, hybrid genome instability can be an interesting mechanism to improve phenotypic diversity, allowing yeast to colonize their environment, in particular stressful/variable industrial environments. Although additional studies on the mechanisms and interactions that modulate complex aneuploid genomes are necessary, it is becoming clearer that genome instability can be an important mechanism for the adaptation to changing and stressful environments.

## Supplementary Data

Supplementary material 1

Supplementary material 2
