# Supplementary material for: Genomic instability in an interspecific hybrid of the genus Saccharomyces: a matter of adaptability
Source: Microb Genom. 2020 Oct 6;6(10):mgen000448. doi: 10.1099/mgen.0.000448 (PMC7660253; doi:10.1099/mgen.0.000448)
Supplement: Supplementary material 2 [file mgen-6-448-s002.pdf]

Supplemental material

Genomic instability in an interspecific hybrid of the Saccharomyces genus: a matter of adaptability.

Miguel Morard[1,2], Clara Ibáñez[2], Ana C. Adam[2], Amparo Querol[2], Eladio Barrio[1,2], Christina Toft[1,2,4*]

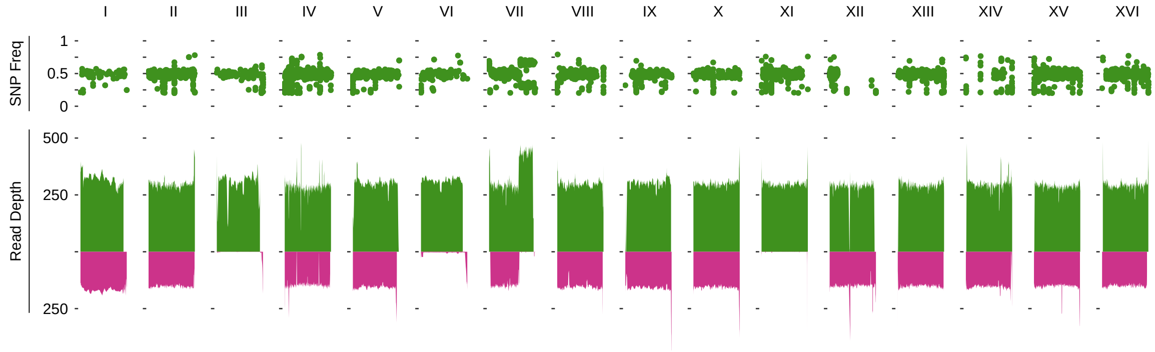

Figure S1.Genome Structure of the strain VIN7. The Read Depth is calculated as the mean of 10kb window moving by 1kb steps. Only heterozygous SNPs in the *Sc* sub-genome are plotted.

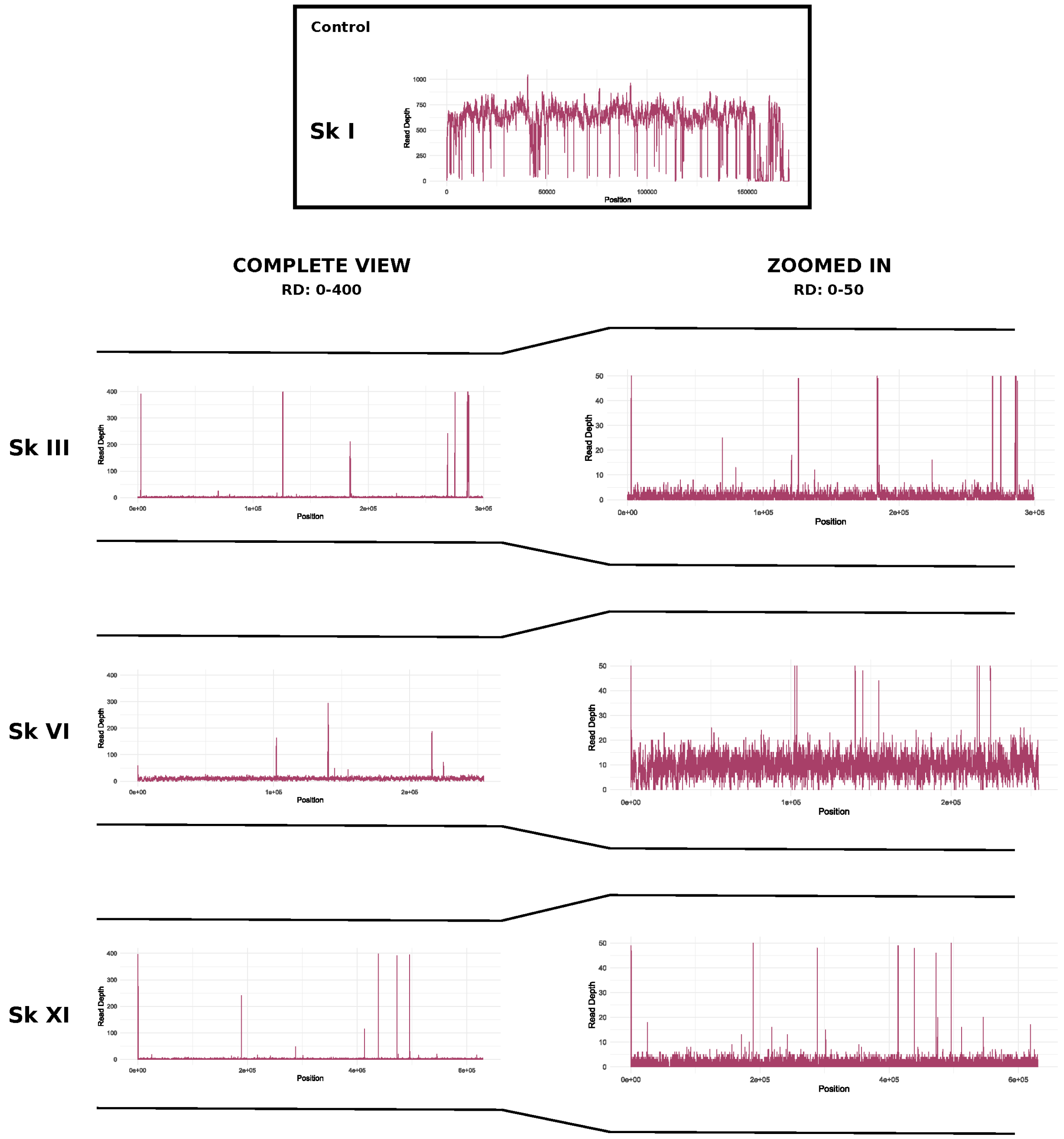

Figure S2. Detecting the low frequency chromosome III,VI and XI of Sk. The Read Depth is plotted for all nucleotide positions in the four chromosomes. Chromosome Sk I was used as the control for the expected coverage of a Sk chromosome. Sk I has a scale of 0-1000 read depth (RD), where the left panel of Sk III, VI and XI is shows a comparative scale to Sk I with 0-400 RD. The right panel is a zoomed on the three chromosomes with a 0-50 RD.

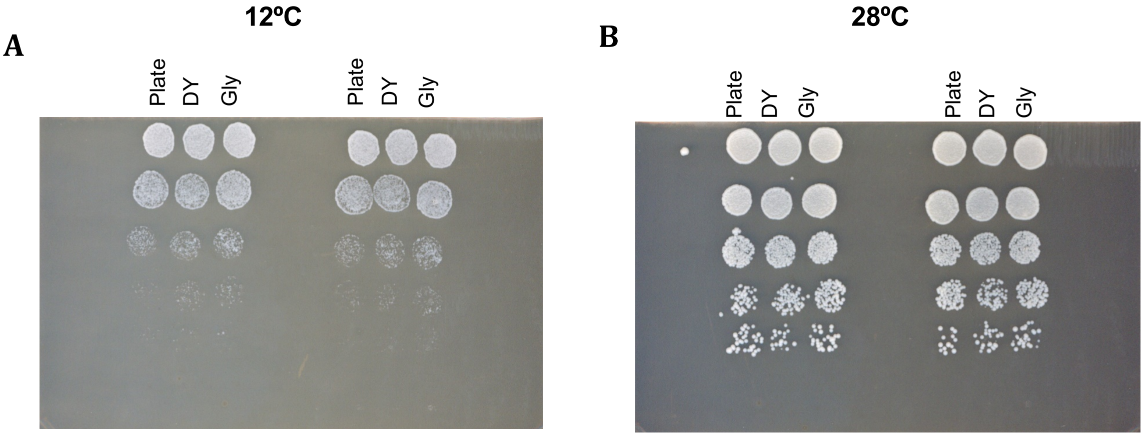

Figure S3. Effect of the temperature on yeast growth phenotypes. After cultivation in GPY liquid medium at 28ºC, VIN7 from dry yeast (DY), glycerinated (Gly) and Plate (Plate) were spotted on solid GPY medium at different dilutions and incubated at 12°C (A) and 28°C (B) for 2 days. The experiments were performed in duplicate.
